# Pigmented purpuric dermatoses versus purpuric mycosis fungoides: red flags from a clinicopathologic case series^؆^

**DOI:** 10.1016/j.abd.2026.501434

**Published:** 2026-08-17

**Authors:** Márcio Fellipe Menezes Viana, Renata Ferreira Magalhães, Juliana Yumi Massuda Serrano, Elisa Nunes Secamilli, Andréa Fernandes Eloy da Costa França

**Affiliations:** Department of Internal Medicine, Division of Dermatology, Faculty of Medical Sciences, Universidade Estadual de Campinas, Campinas, SP, Brazil

Dear Editor,

Pigmented Purpuric Dermatoses (PPD) are chronic purpuric eruptions characterized by erythrocyte extravasation and dermal hemosiderin deposition, usually without true vasculitis.^1^, ^2^ Some PPD variants may show a subtle lichenoid/band-like lymphocytic pattern, which can blur the differential diagnosis when interpreted without clinicopathologic correlation.^3^ In clinical practice, PPD may overlap clinically and histologically with early cutaneous T-Cell Lymphoma (CTCL), particularly purpuric Mycosis Fungoides (MF), a rare variant that can closely mimic PPD.^4^, ^5^, ^6^, ^7^, ^8^ Because MF requires staging and CTCL-directed management, distinguishing these entities remains clinically relevant and is a frequent diagnostic pitfall.^9^, ^10^

We report a clinicopathologic series of five PPD cases (ages 14–72 years) and two cases of purpuric MF, highlighting practical “red flags” that should prompt Immunohistochemistry (IHC), repeat biopsies, and longitudinal reassessment.

In our series, the PPD spectrum comprised Majocchi's purpura, lichen aureus, Schamberg disease, Gougerot-Blum pigmented purpuric lichenoid dermatitis, and Doucas-Kapetanakis eczematid-like purpura (Fig. 1, Fig. 2). Lesions were predominantly chronic, flat, and preferentially involved the lower limbs. Histopathology showed superficial chronic perivascular dermatitis with erythrocyte extravasation and variable hemosiderin deposition, without vasculitis. Notably, two PPD subtypes (lichen aureus and Majocchi) displayed a subtle band-like lymphocytic pattern, a recognized feature that can blur the differential diagnosis if interpreted without clinicopathologic correlation.^1^, ^2^, ^3^

**Fig. 1:**
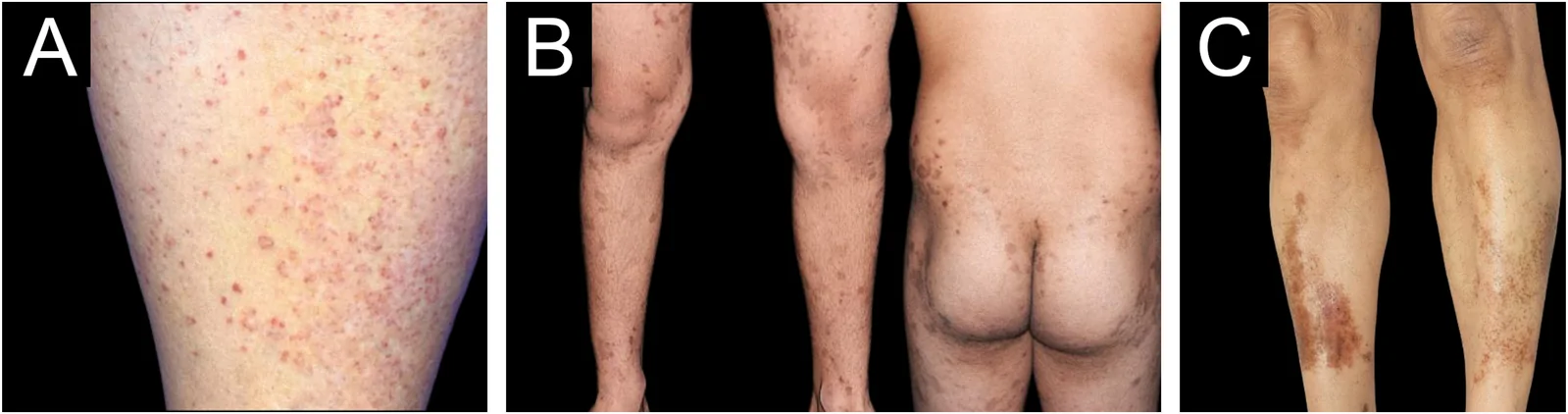
Representative clinical features of Pigmented Purpuric Dermatoses (PPD) subtypes in this series: (A) Majocchi's purpura ‒ annular erythematoviolaceous lesions with telangiectasias on the lower limb. (B) Lichen aureus ‒ multiple brownish macules on the lower back, buttocks, and lower limbs. (C) Schamberg disease ‒ brownish-orange macules on the lower limbs with a “cayenne pepper” speckled pattern.

**Fig. 2:**
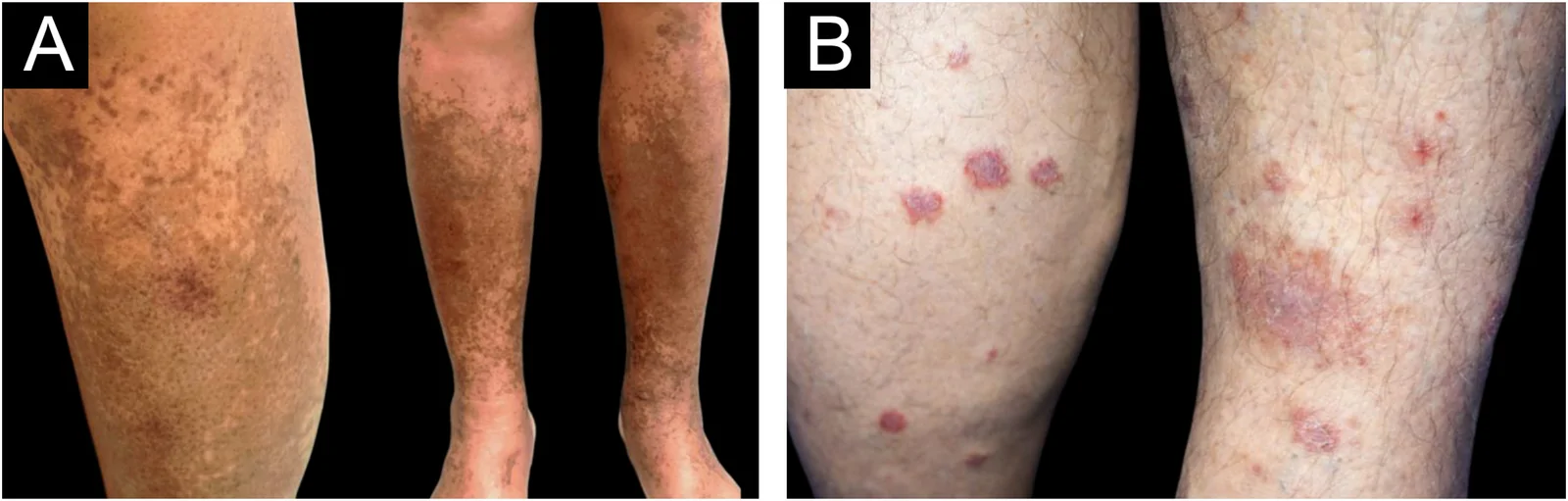
Representative clinical features of Pigmented Purpuric Dermatoses (PPD) subtypes in this series: (A) Gougerot-Blum pigmented purpuric lichenoid dermatitis ‒ coalescing brownish papules and macules with purpuric and lichenoid areas on the lower limbs. (B) Doucas-Kapetanakis eczematid-like purpura ‒ annular erythematoviolaceous eczematous plaques with a purpuric center and peripheral scale on the lower limbs.

Two patients had purpuric MF. Case MF-1 presented with persistent brownish macules and erythematous scaly patches involving the legs, buttocks, arms, and palmoplantar areas, associated with petechiae (Fig. 3). Biopsy demonstrated a superficial band-like lymphocytic infiltrate, papillary dermal fibroplasia, focal spongiosis, and epidermotropism. IHC showed frequent CD3+ lymphocytes in the dermis and epidermis with CD7 hypoexpression, supporting early-stage MF.

**Fig. 3:**
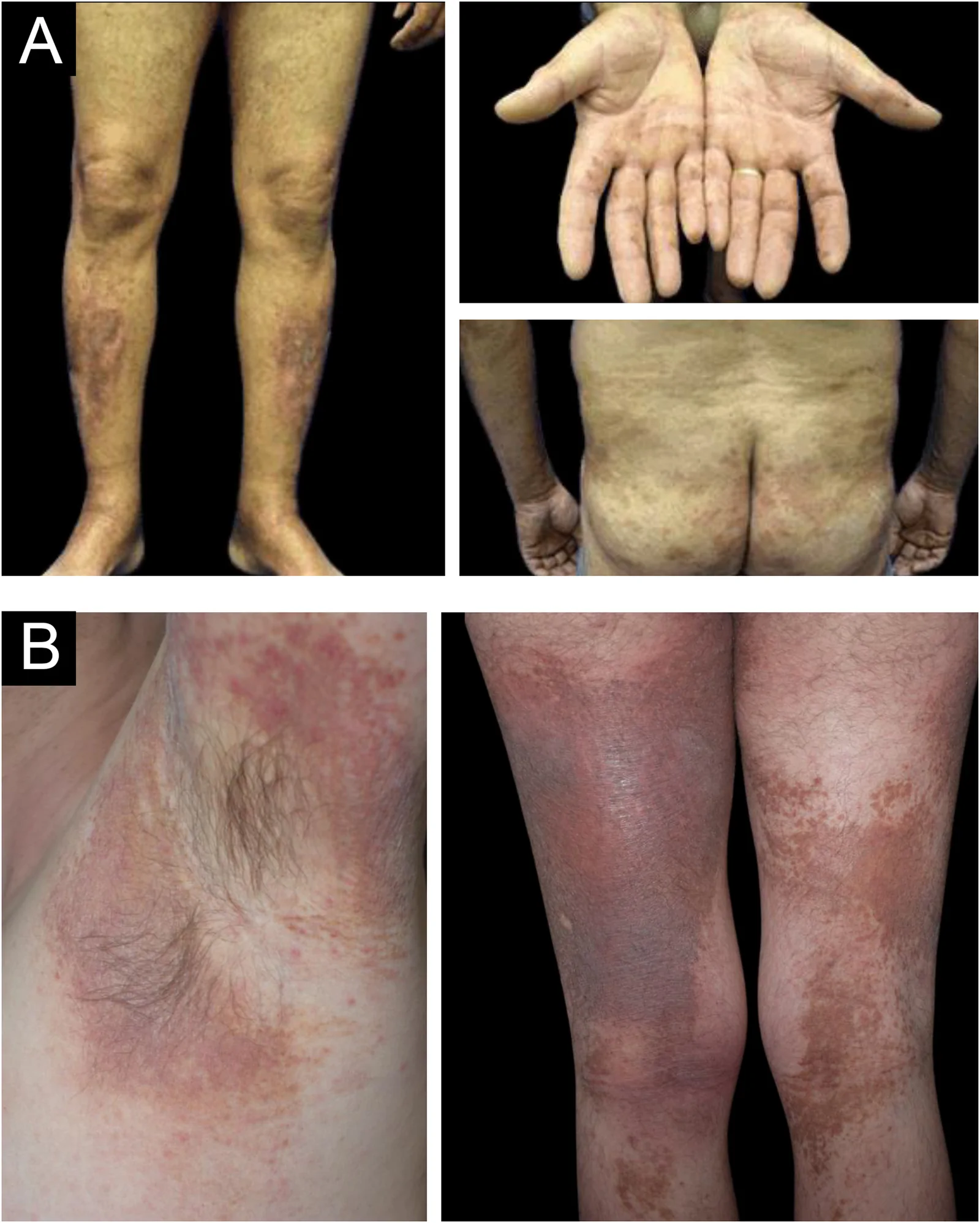
Clinical presentation of purpuric Mycosis Fungoides (MF): (A) MF-1 – brownish macules and erythematous scaly patches on the legs, buttocks, arms, and palmoplantar areas, with petechiae. (B) MF-2 – violaceous patches with mild scale and discrete perilesional erythema involving mainly the lower limbs/feet and left abdomen, with a more purpuric aspect in friction/contact areas (hips/waistband).

Case MF-2 presented with violaceous patches with mild scale and discrete perilesional erythema, involving mainly the lower limbs/feet and the left abdomen, with a more purpuric aspect in friction/contact areas (hips/waistband) and the left axilla (Fig. 3). Multiple biopsies (left abdomen papule and scaly plaques; posterior left thigh) showed a superficial lymphocytic infiltrate variably accentuated at the dermoepidermal junction and, in the thigh, forming a dense band-like subepidermal infiltrate with focal epidermotropism, associated with fibroplasia, marked erythrocyte extravasation, and significant dermal hemosiderosis (Perls-positive in the thigh specimen). IHC demonstrated a predominance of T cells (CD2+/CD3+/CD5+) with CD4 predominance, marked CD7 hypoexpression, low proliferative index (Ki-67 < 10%), and no aberrant CD30 expression (CD30 negative; only rare CD56+ cells), supporting early-stage MF (Fig. 4). Treatment included methotrexate and phototherapy. A subsequent biopsy again showed epidermotropic/folliculotropic lymphocytes with prominent erythrocyte extravasation and a wedge-shaped superficial infiltrate. In this specimen, IHC revealed a focal subset of larger CD30+ cells (approximately 20% in a focal area; approximately 10% among small-to-medium cells) with increased proliferation (Ki-67 approximately 60%‒70%); however, no morphologic criteria for large-cell transformation were identified.

**Fig. 4:**
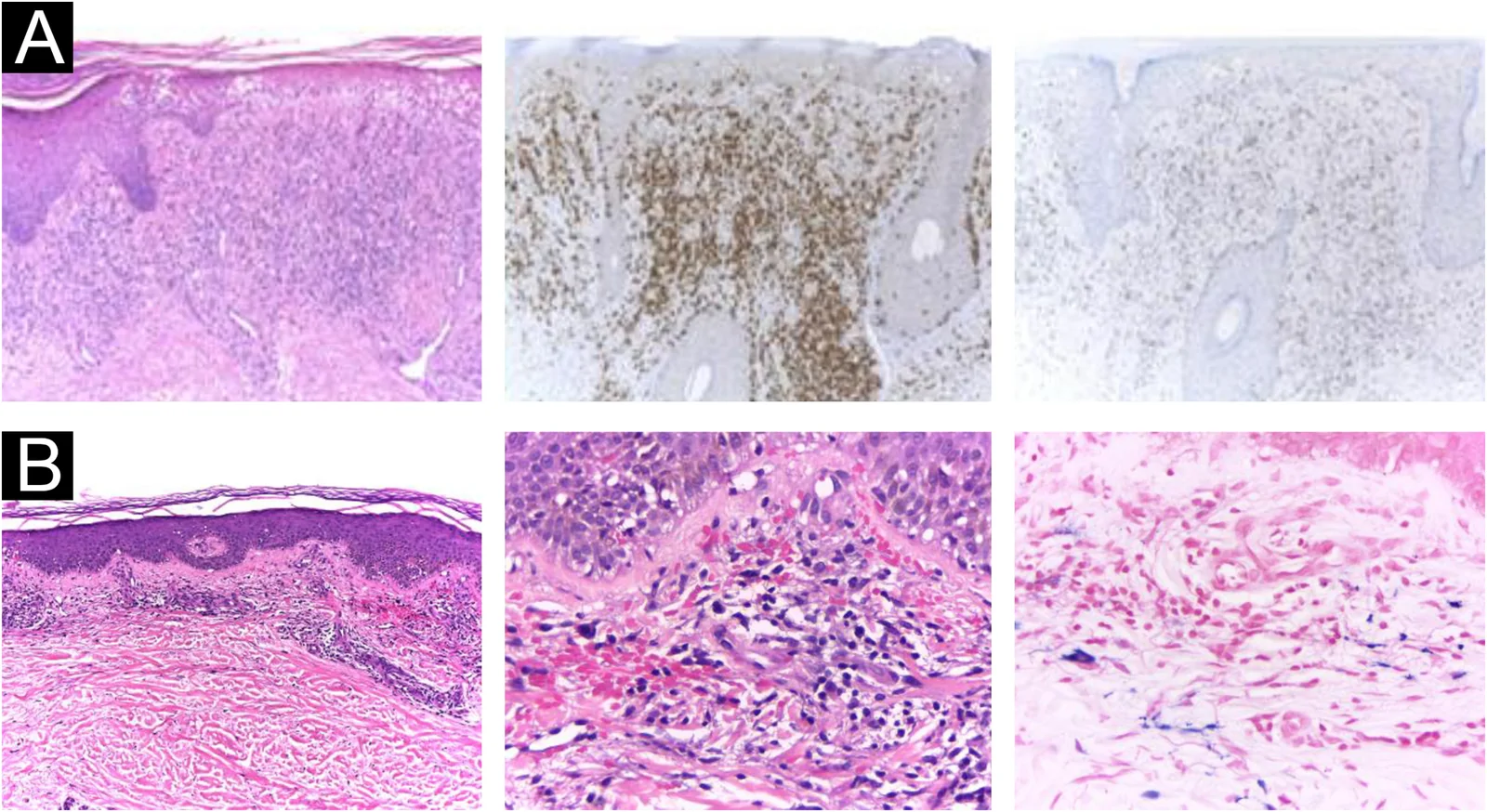
Histopathologic comparison supporting the differential diagnosis. (A) Purpuric MF (MF-1): superficial band-like lymphocytic infiltrate with papillary dermal fibroplasia, focal spongiosis, and epidermotropism (Hematoxylin & eosin). IHC shows frequent CD3+ lymphocytes in the dermis and epidermis and along the follicular infundibulum, with CD7 hypoexpression. (B) PPD: superficial perivascular (and mildly interstitial) chronic dermatitis with erythrocyte extravasation and hemosiderin, without true vasculitis (Hematoxylin & eosin).

Distinguishing PPD from purpuric MF is among the most challenging differentials within purpuric dermatoses, particularly in early disease. Based on our cases, key red flags for MF in a patient initially suspected of PPD include: (i) Evolution to scaly patches/plaques or subtle induration; (ii) Involvement of unusual sites (buttocks, palmoplantar areas, or intertriginous/friction zones); (iii) New-onset or worsening pruritus; (iv) Polymorphic lesions in the same patient; (v) Lack of sustained improvement despite adequate and adherent topical therapy, particularly when accompanied by lesion progression or additional red flags; (vi) Prominent band-like infiltrate with epidermotropism/folliculotropism; and (vii) Loss/hypoexpression of pan-T markers such as CD7.^3^, ^4^, ^5^, ^6^, ^7^, ^8^

Because subtle band-like patterns may also occur in PPD subtypes, diagnosis should rely on robust clinicopathologic correlation rather than a single histologic feature. Serial biopsies may be required not only to document diagnostic findings (epidermotropism and loss of pan-T markers), but also because histologic and immunophenotypic profiles can evolve over time, occasionally showing focal CD30 expression without overt large-cell transformation.^3^, ^4^, ^5^, ^6^, ^7^, ^8^ This reinforces the need for reassessment when the clinical course is atypical.

## Authors' contributions

Márcio Fellipe Menezes Viana: Approval of the final version of the manuscript; data collection, analysis and interpretation; preparation and writing of the manuscript; study conception and planning.

Renata Ferreira Magalhães: Approval of the final version of the manuscript; data collection, analysis and interpretation; manuscript critical review.

Juliana Yumi Massuda Serrano: Approval of the final version of the manuscript; data collection, analysis and interpretation; study conception and planning.

Elisa Nunes Secamilli: Approval of the final version of the manuscript; manuscript critical review.

Andréa Fernandes Eloy da Costa França: Approval of the final version of the manuscript; effective participation in research orientation; manuscript critical review; study conception and planning.

## Financial support

This study was funded exclusively by the authors, with no financial support from public or private institutions.

## Research data availability

Does not apply.

## Conflicts of interest

None declared.
